# Synergy between chemotherapy and cancer vaccination

**DOI:** 10.18632/aging.100752

**Published:** 2015-05-30

**Authors:** Tetje C. van der Sluis, Sjoerd H. van der Burg, Cornelis J.M. Melief

**Affiliations:** Department of Immunohematology and Blood Transfusion, Leiden University Medical Centre (LUMC), the Netherlands

Cancer immunotherapy has become a powerful treatment option for a wide range of cancers. The goal of most cancer immunotherapies is to activate cancer-specific T lymphocytes that can recognize and kill tumor cells expressing tumors-specific antigens. Examples of these tumor-antigens are viral antigens, mutated antigens, and overexpressed self-antigens. Since central tolerance is exclusively induced for self-antigens in the thymus, mutated (neo-)antigens and viral antigens are ideal targets for therapeutic cancer vaccination.

Human Papilloma Virus (HPV) is an example of a virus that is clearly associated with several cancers, the most frequent of which are cervical cancer and oropharyngeal cancer. HPV has two oncogenes (E6 and E7) that are critical for the induction and maintenance of the transformed cell stage and are constitutively expressed by the cancer cells, making them ideal targets for therapeutic vaccination. Our vaccination approach involves immunization with overlapping synthetic long peptides (SLP) of the oncogenes E6 and E7. This vaccination strategy induces potent T cell responses associated with complete regressions of tumors in mice, and in patients with premalignant lesions [1]. However, in patients with advanced and recurrent tumors, the immune response to the vaccine is much weaker and not inducing a clinical effect. This raised the question how this vaccination could be improved.

In a preclinical mouse model for HPV-induced cancers we tested for seven clinically relevant chemotherapeutics whether they could be combined with SLP vaccination [2]. Importantly, none of the tested chemotherapeutics impaired the immune response to SLP vaccination, and four of them significantly improved vaccination-related survival. The combination of the DNA cross-linking agent cisplatin and SLP vaccination most effectively improved long term survival. In depth analysis of this synergy revealed that HPV-specific CD8 T cells were crucial for the observed synergy. Analysis of the vaccine-induced tumor-infiltrating CD8 T cells showed that a large proportion of these cells produced interferon-gamma (IFN-γ) as well as tumor necrosis factor α (TNFα) upon recognition of HPV antigen. Furthermore, the tumors of mice treated with both cisplatin and peptide vaccination contained lower numbers of proliferating tumor cells and an increased number of apoptotic tumor cells compared to untreated or single agent treated tumors. Notably, we found that a combined effect of TNFα and cisplatin causes enhanced apoptosis of the tumor cells. Neutralization of TNFα by monoclonal antibody injection of mice treated with cisplatin and SLP vaccination resulted in a decreased overall survival when compared to control mice. Together, these data indicate that TNFα is critical for the synergy between vaccination and cisplatin treatment [2].

**Figure 1:**
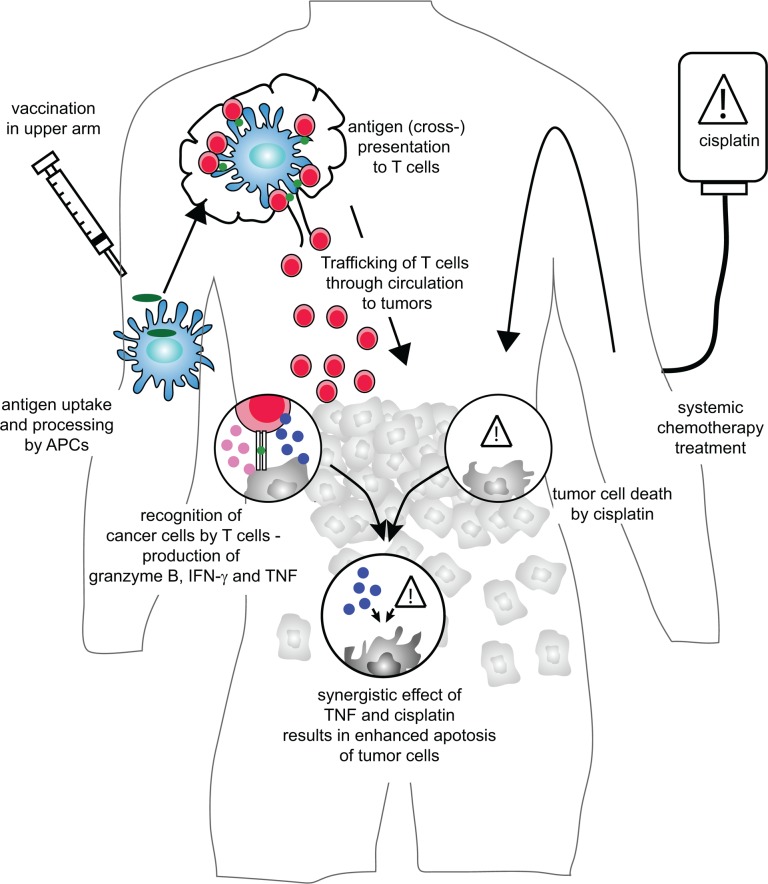
Coordinated action of combined treatment with chemotherapy and vaccination When a cancer patient is vaccinated with synthetic long peptides, these peptides are taken up, processed and presented by antigen presenting cells to T cells in the lymph node. These T cells proliferate and travel to the tumor where they recognize tumor antigen and produce effector molecules such as Granzyme B, IFN‐γ (pink circles) and TNFα (blue circles). At the same time, systemic chemotherapy treatment results in tumor cell death. Both individual treatments are rarely sufficient to cause complete tumor eradication. However, the combined action of TNFα and chemotherapy may result in synergistic cell death of tumor cells, resulting in enhanced survival of tumor bearing individuals.

TNFα is an important member of the “TNF superfamily”, a group of molecules that each bind to their corresponding ligands, all members of the “TNF receptor superfamily”. Triggering of the receptors can result in either activation and proliferation or apoptosis of the target cell. For example, TNFα is involved in the activation of macrophages and endothelial cells, the development of tumors but also in tumor cell death. The coordinated action between TNFα and chemo-therapeutics is in itself not unexpected. Other members of the TNF family synergize with various chemo-therapeutics to cause tumor cell death. Additionally, TNFα itself is strongly associated with cisplatin induced nephrotoxicity [3], and the combination of doxorubicin or melphalan and TNFα in isolated limb perfusion can be extremely efficient as well [4]. However, the serious side effects of systemic treatment with TNFα limit its clinical use and intratumoral administration remains challenging [5]. Importantly, our data indicate that T cells – systemically induced by vaccination - can travel into tumors and locally produce enough TNFα in close proximity to tumor cells to synergize with cisplatin. This not only overcomes the need for the maximum tolerated dose of cisplatin but also avoids the toxic effects of systemic TNFα but still permits the cooperation between TNFα and cisplatin (Figure 1).

These data are of particular interest for the field of cancer immunotherapy. Recent studies have shown that neo-antigen-specific T cells can be successfully mobilized by vaccination with long peptides, and that these T cells are capable of producing pro-inflammatory cytokines [6;7]. These data indicate that cancer treatment via peptide vaccination could be broadly applied, and might provide clinical benefit in many more cancer types. Checkpoint blockade therapy such as delivery of monoclonal antibodies against PD-1 and CTLA-4 can also enhance the number of TNFα producing intratumoral T cells [7]. Together these data indicate that enhancement of intratumoral TNFα levels via T cell based immunotherapy is feasible and attractive via different methods. The combination of various T cell based immunotherapies with cisplatin might boost the anti-tumor responses via mechanisms dependent on the interaction between TNFα and cisplatin. It will be of interest to explore whether this is indeed what happens when different T cell-based immunotherapies are combined clinically with cisplatin.
